# Efficacy of BRAF and MEK Inhibition in Patients with BRAF-Mutant Advanced Melanoma and Germline CDKN2A Pathogenic Variants

**DOI:** 10.3390/cancers13102440

**Published:** 2021-05-18

**Authors:** Francesco Spagnolo, Bruna Dalmasso, Enrica Tanda, Miriam Potrony, Susana Puig, Remco van Doorn, Ellen Kapiteijn, Paola Queirolo, Hildur Helgadottir, Paola Ghiorzo

**Affiliations:** 1 Medical Oncology 2, IRCCS Ospedale Policlinico San Martino, 16132 Genoa, Italy; francesco.spagnolo@hsanmartino.it (F.S.); enricateresa.tanda@hsanmartino.it (E.T.); 2 IRCCS Ospedale Policlinico San Martino, Genetics of Rare Cancers, 16132 Genoa, Italy; brunasamia.dalmasso@dimi.unige.it; 3 Genetics of Rare Cancers, Department of Internal Medicine and Medical Specialties, University of Genoa, 16132 Genoa, Italy; 4 Melanoma Unit, Dermatology Department, Hospital Clinic de Barcelona, Institut d’Investigacions Biomèdiques August Pi i Sunyer (IDIBAPS), Universitat de Barcelona, 08007 Barcelona, Spain; potrony@clinic.cat (M.P.); spuig@clinic.cat (S.P.); 5 Centro de Investigación Biomédica en Red (CIBER) de Enfermedades Raras, Instituto de Salud Carlos III, 08036 Barcelona, Spain; 6 Department of Dermatology, Leiden University Medical Center, 2333 Leiden, The Netherlands; R.van_Doorn@lumc.nl; 7 Department of Medical Oncology, Leiden University Medical Center, 2333 Leiden, The Netherlands; h.w.kapiteijn@lumc.nl; 8 Melanoma, Sarcoma & Rare Tumors Division, European Institute of Oncology (IEO), 20141 Milan, Italy; paola.queirolo@ieo.it; 9 Department of Oncology Pathology, Karolinska Institutet and Karolinska University Hospital Solna, 171 64 Stockholm, Sweden; hildur.helgadottir@sll.se

**Keywords:** melanoma, CDKN2A, targeted therapy, BRAF inhibitors, MEK inhibitors, genetic counseling, melanoma susceptibility

## Abstract

**Simple Summary:**

In our study, we retrospectively collected data of patients with germline CDKN2A pathogenic variants who received targeted therapy for advanced melanoma across four European centers. Since loss of CDKN2A function may intrinsically limit the activity of MAPK-directed targeted therapy, we decided to assess whether patients with germline CDKN2A pathogenic variants may achieve suboptimal results with BRAF and MEK inhibitors. To the best of our knowledge, this is the first study reporting on patients with BRAF-mutant advanced melanoma and a germline CDKN2A pathogenic variant who received treatment with BRAF with or without MEK inhibitors. Despite the limitations of our study, mostly due to the rare frequency of CDKN2A pathogenic variants, a challenge for the conduction of prospective trials with proper sample size, our results support treatment with targeted therapy in this subset of patients.

**Abstract:**

Inherited pathogenic variants (PVs) in the CDKN2A tumor suppressor gene are among the strongest risk factors for cutaneous melanoma. Dysregulation of the p16/RB1 pathway may intrinsically limit the activity of MAPK-directed therapy due to the interplay between the two pathways. In our study, we assessed, for the first time, whether patients with germline CDKN2A PVs achieve suboptimal results with BRAF inhibitors (BRAFi)+/−MEK inhibitors (MEKi). We compared the response rate of nineteen CDKN2A PVs carriers who received first-line treatment with BRAFi+/−MEKi with an expected rate derived from phase III trials and “real-world” studies. We observed partial response in 16/19 patients (84%), and no complete responses. The overall response rate was higher than that expected from phase III trials (66%), although not statistically significant (*p*-value = 0.143; 95% CI = 0.60–0.97); the difference was statistically significant (*p*-value = 0.019; 95% CI = 0.62–0.97) in the comparison with real-world studies (57%). The clinical activity of BRAFi+/−MEKi in patients with germline CDKN2A PV was not inferior to that of clinical trials and real-world studies, which is of primary importance for clinical management and genetic counseling of this subgroup of patients.

## 1. Introduction

Inherited pathogenic variants (hereafter named PVs or mutations) in the CDKN2A tumor suppressor gene, which encodes for the cell cycle inhibitors p16ink4A and p14ARF, constitute the main risk factor for individuals with an inherited predisposition to melanoma, who are at increased risk of developing multiple melanomas and other tumors, in particular pancreatic cancer [1]. Few studies have addressed the role of germline CDKN2A PVs in survival and response to therapy. Indeed, the impact of these variants on overall survival (OS) and melanoma-specific survival (MSS) is controversial. In a previous study, CDKN2A PV carriers were reported to have inferior MSS as compared with melanoma cases with no CDKN2A mutations (hazard ratio [HR] = 2.50, 95% confidence interval [CI] = 1.49 to 4.21) that was independent of stage, age and sex, and not associated with the diagnosis of subsequent primary melanomas or other tumors [2]. However, in a high mutation-prevalence cohort of melanoma patients undergoing a mutation-based follow-up, no differences were found between CDKN2A-positive and CDKN2A-negative patients in terms of OS (HR = 0.85; 95% CI = 0.48–1.52) and MSS (HR = 0.86; 95% CI = 0.38–1.95) [3].

Somatic CDKN2A alterations are common driver events in melanoma, and are associated with tumor proliferation, increased risk of metastases and decreased OS [4]. In general, cutaneous melanomas have a very high mutation burden, which has been associated with improved response to immunotherapy with immune checkpoint inhibitors and adoptive cell therapies, and there is growing evidence that it may be an independent predictive factor for efficacy of immunotherapy [5]. In a retrospective study of 19 metastatic melanoma patients with germline CDKN2A mutations, with 11 of the 19 carriers (58%) responding to immunotherapy, it was found that response to immunotherapy was significantly higher than that observed in clinical trials (*p* = 0.03, binomial test against an expected rate of 37%); a higher rate of complete responses was also observed, with six of the 19 carriers (32%) achieving a complete response (*p* = 0.01, binomial test against an expected rate of 7%) [5]. A plausible underlying mechanism is that melanomas with somatic CDKN2A mutations have a significantly higher total number of mutations compared with CDKN2A somatic mutation-negative melanomas [5].

**Figure 1:**
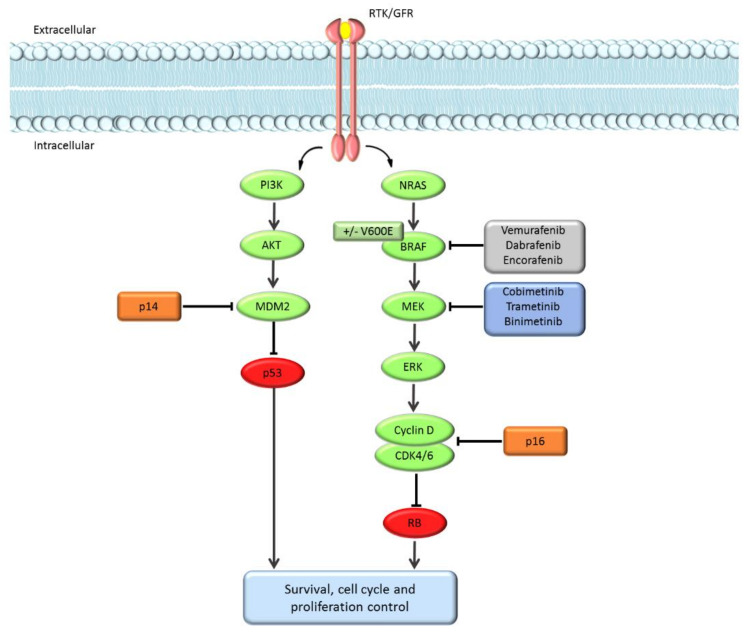
Interplay between the mitogen-activated protein kinase (MAPK) and p16/p14 regulated pathways. ERK signaling is regulated by extracellular signals binding to receptor tyrosine kinases (RTKs). Activated RTKs promote RAS-mediated dimerization of RAF; RAF dimers phosphorylate and activate MEK1/2, which in turn phosphorylate and activate ERK1/2. Activated ERK promotes proliferation, e.g., by activation of the Cyclin D and CDK4/6 complex that inhibits the tumor suppressor RB1. P16 prevents proliferation by negatively regulating Cyclin D1/CDK4 function. In BRAF-mutated cells, BRAFV600E is constitutively active as a monomer, leading to high ERK signaling. BRAF and MEK blockade effectively inhibit ERK signaling. However, dysregulation of the p16/RB1 pathway may sustain tumor growth regardless of BRAF/MEK inhibition and may confer resistance to treatment. Another mechanism of resistance to BRAF/MEK inhibition is through activation of the PI3K-AKT pathway that promotes cell survival and proliferation, e.g., by the activation of MDM2 protein which inhibits the tumor suppressor p53. P14 prevents such proliferation by negatively regulating MDM2.

Besides immunotherapy, the emergence of MAPK-directed targeted therapy has revolutionized the melanoma oncology field in the last years. The identification of BRAF V600 somatic mutations in approximately 50% of cutaneous melanomas [6] led to the development of highly active MAP kinase small molecule inhibitors. First, the BRAF inhibitors (BRAFi) vemurafenib and dabrafenib were approved as single agents for the treatment of BRAF-mutated advanced melanoma [7]. Then, four randomized phase III trials demonstrated the superiority, in terms of efficacy, of combined BRAFi and MEK inhibition (MEKi) over treatment with single-agent BRAFi [7], and combination therapy was approved by the regulatory agencies. However, about one third of patients treated with targeted therapy do not achieve tumor regression because of intrinsic/primary resistance, and most patients who respond to therapy ultimately develop acquired/secondary resistance, leading to progressive disease. Dysregulation of the p16/RB1 or p14ARF/MDM2/p53 pathways may limit the activity of MAPK-directed targeted therapy [8] (Figure 1), and CDKN2A loss in the tumor was an independent predictor of shorter PFS BRAF-mutant metastatic melanoma patients treated in a study with the BRAFi dabrafenib as a single agent [9]. Moreover, in a phase III study of dabrafenib in combination with the MEKi trametinib, somatic CDKN2A mutations were associated with shorter PFS, with 6% of patients with a CDKN2A mutation being alive and free of disease progression at three years versus 27% of mutation-negative patients [10].

Previous studies have shown that CDKN2A germline PVs does not affect the prevalence of somatic BRAF and NRAS mutations in cutaneous melanomas [11], and that familial and sporadic melanomas share similar gene expression signatures [12]. However, so far, no studies have addressed the effects of MAPK-directed targeted therapies in patients with BRAF-mutant metastatic melanoma and germline CDKN2A PVs.

## 2. Materials and Methods

Nineteen CDKN2A mutation carriers who developed BRAF-mutant metastatic melanoma and underwent first-line treatment with BRAFi alone or in combination with MEKi were identified by reviewing medical records of carriers enrolled in follow-up studies for familial melanoma in Sweden, the Netherlands, Italy and Spain. The different studies in which carriers were identified have been described previously [13,14,15,16,17,18]. The efficacy of second-line immunotherapy in eight of the patients in this cohort was reported in a previous study [5].

Data collected included the type of germline CDKN2A pathogenic variant, sex and age at start of first-line treatment, tumor stage (according to the eighth edition of the AJCC cancer staging system, implemented in January 2018), type of targeted therapy, responses, PFS, OS and emergence of severe treatment-related side effects. CDKN2A mutation carriers received treatments according to standard dosage and treatment schedules. Patients were divided in two groups depending on whether they underwent treatment with a BRAFi as a single agent or in combination with a MEKi. One patient received an anti-PD-1 drug in addition to BRAFi + MEKi as part of a clinical trial.

The best response achieved was assessed in the CDKN2A PV carriers and overall response rate (ORR) was compared with responses reported in phase III clinical trials (Table S2) and “real-world” studies (Table S3). By a binomial test, we evaluated if there was a different ORR in CDKN2A PV carriers compared with an expected rate. The expected rate was calculated as a median of the ORRs in the clinical trials and “real world” studies, respectively, weighted against the number of carriers in our study receiving each type of therapy. All tests were two-sided and were conducted within the R environment for Statistical computing [19].

## 3. Results

Patients’ characteristics are summarized in Table 1, while individualized data are listed in Table S1. Among the 19 identified CDKN2A PV carriers (10 men and nine women), median age at the start of treatment was 57 years (range 29–69 years), similar to the age at treatment start reported in phase III trials of BRAFi and BRAFi + MEK i (Table S2) and real-world studies (Table S3).

Eleven patients (58%) had stage M1c disease and five (26%) had brain metastases at baseline (M1d), while none had unresectable stage III disease. This cohort of patients appeared to have worse prognostic features than those enrolled in phase III trials of BRAFi and BRAFi + MEKi (see Table S2). However, the prognostic features of the CDKN2A mutated patients in the study were more similar to those reported in the real-world studies (see Table S3). Sixteen patients (84%) achieved a partial response (PR), no complete responses (CR) were seen. The ORR of 84% was numerically higher than that expected from phase III trials (66%), but the binomial test was not statistically significant (*p*-value = 0.143; 95% CI = 0.60–0.97); the difference was statistically significant (*p*-value = 0.019; 95% CI = 0.60–0.97) when the ORR of our cohort was compared with the expected response rate calculated from real-world studies (57%). All patients in our study received BRAFi + MEKi as a first-line treatment, and this was also the case in all but one of the phase 3 studies, whereas all the real-world studies included both untreated and previously treated patients.

Among the twelve CDKN2A PV carriers who underwent combined treatment with BRAFi + MEKi, seven (58%) had stage M1c disease and three (25%) had brain metastases at baseline. This cohort of patients also had worse prognostic features than those enrolled in phase III trials of BRAFi + MEKi (see Supplementary Table S2). All patients achieved PR and in this subset of patients, the ORR (100%) was significantly higher than that expected from both phase III trials, 69.6% (*p*-value = 0.02327; 95% CI = 0.74–1.00) and real-world studies, 68.6% (*p*-value = 0.02327; 95% CI = 0.74–1.00). The overall and progression-free survival (PFS) in months for each patient is shown in Supplementary Table S1. Median PFS of CDKN2A PV carriers was 6.0 months and median OS was 13.0 months. In patients receiving single BRAFi (*n* = 7) median PFS was 3.0 months, while it was 9.5 months in patients receiving BRAFi + MEKi (*n* = 12). To compare, in clinical trials where single vemurafenib or dabrafenib were administered, median PFS was 6.9 and 5.1 months, respectively, whereas in those with dabrafenib + trametinib or encorafenib + binimetinib combinations, PFS was 11.1 and 14.9 months, respectively (Table S2).

## 4. Discussion

To the best of our knowledge, this is the first study reporting on patients with BRAF-mutant advanced melanoma and a germline CDKN2A PV who received treatment with BRAFi, with or without MEKi.

As dysregulation of the p16/RB1 or p14ARF/MDM2/p53 pathways may intrinsically limit the activity of BRAFi in advanced melanoma, we hypothesized that our cohort of patients with germinal CDKN2A PV may achieve suboptimal results with targeted therapy, especially those who received a BRAFi as a single agent. Conversely, anti-tumor response rates were higher in our cohorts compared with phase III and real-world clinical studies, even though our patients showed worse prognostic features (such as brain metastases). The binomial test, not statistically significant for the comparison with phase III studies, was significant for the comparison with real-world studies. Noteworthy, patients in our study cohort had all received first-line treatment, while those enrolled in the real-world studies were both untreated and previously treated patients, and previous treatment lines is a factor known to be associated with poorer outcomes. No CRs were observed in our cohort, while 5–7% of patients achieved CR in studies with BRAFi as single agents and 13–21% in case of combination treatment. Further, median PFS, both for CDKN2A PV carriers treated with single-agent BRAFi and those treated with BRAFi + MEKi, was numerically shorter than that reported in clinical trials. Although the PFS difference needs to be interpreted with caution, the updated results of phase III clinical trials with dabrafenib and trametinib showed a strong association between CR and long-term survival: 49% of patients who achieved a CR were alive and free of disease progression at 5 years versus 16% of patients with PR [20]. The lack of CRs in our cohort of patients may therefore have resulted in shorter PFS.

In addition to the high proportion of patients with poor baseline prognostic features, a possible explanation to the lack of CRs and shorter median PFS in the patients included in this study may be that the dysregulation of the p16/RB1 or p14ARF/MDM2/p53 pathways sustained by CDKN2A pathogenic mutations, as well as the high tumor mutational load in CDKN2A-mutant melanomas, may limit the chances to obtain a full and lasting inhibition of the oncogenic pathways sustaining tumor cell proliferation. This result is in contrast with that observed in patients with germline CDKN2A variants who received immune-checkpoint inhibitors, as in the case of immunotherapy, the high mutational burden may favor anti-tumor responses [5].

Our study has some limitations, mainly due to the retrospective design and small sample size. However, germline CDKN2A variants are rare, and only a subset of CDKN2A positive patients with primary melanoma ultimately develop distant metastases and need systemic therapy. In addition to that, some patients with germline CDKN2A PV may not undergo genetic testing, so clinicians may be unaware of this information when they start first line treatment for advanced melanoma.

## 5. Conclusions

In summary, our analysis showed that the ORR of BRAFi and MEKi in patients with BRAF-mutant advanced melanoma and germline CDKN2A PVs was not inferior to that observed in clinical trials and real-world studies, which we believe to be a relevant information for clinicians who manage CDKN2A PV carriers with BRAF-mutant melanoma. Our results, together with those of our previous study on patients receiving immunotherapy [5], are reassuring with regards to the medical treatment of melanoma patients carrying germline CDKN2A PV, and we believe this is a valuable and novel addition to the genetic counseling of these patients.

## Figures and Tables

**Table 1:** Characteristics of the patients with germline *CDKN2A* pathogenic variants and BRAF V600 mutated metastatic melanoma treated with BRAF and MEK inhibitors.

Patients’ Characteristics	Cohort 1 (BRAFi) *n* = 7	Cohort 2 (BRAFi + MEKi) *n* = 12	All Patients *n* = 19
Gender, *n* (%)			
Male	5 (71%)	5 (42%)	10 (53%)
Female	2 (29%)	7 (58%)	9 (47%)
Age, years Median (range)	54 (29–69)	58 (34–69)	57 (29–69)
AJCC 8th edition stage, *n* (%)			
M0	0 (0%)	0 (0%)	0 (0%)
M1a	0 (0%)	2 (17%)	2 (11%)
M1b	1 (14%)	0 (0%)	1 (5%)
M1c	4 (57%)	7 (58%)	11 (58%)
M1d	2 (29%)	3 (25%)	5 (26%)
Baseline LDH *, *n* (%)			
Normal	0 (0%)	3 (25%)	3 (33%)
Elevated	2 (100%)	4 (75%)	6 (67%)
Unknown	5	5	10
Best response, *n* (%)			
Complete response	0 (0%)	0 (0%)	0 (0%)
Partial response	4 (57%)	12 (100%)	16 (84%)
Stable disease	1 (14%)	0 (0%)	1 (5%)
Progressive disease	2 (29%)	0 (0%)	2 (11%)
PFS, median (range) Median PFS (months)	3.0 (2–30)	9.5 (3–30)	6.0 (2–30)

* rate of patients with normal/elevated LDH were calculated only from patients with known data. Abbreviations = LDH: Lactate dehydrogenase, PFS: Progression free survival, BRAFi: BRAF inhibitors, MEKi: MEK inhibitors.

## Data Availability

All data relevant to the study are included in the article or uploaded as supplementary information.

## Supplementary Materials

The following are available online at https://www.mdpi.com/article/10.3390/cancers13102440/s1, Table S1: Individualized data for the cohort of patients with germline CDKN2A pathogenic variants and BRAF V600 mutated metastatic melanoma treated with BRAF and MEK inhibitors included in our study; Table S2: Summary of data from phase III clinical trials on patients with metastatic melanoma treated with BRAF and MEK inhibitors, Table S3: Summary of data from “real world” studies of patients with metastatic melanoma treated with BRAF and MEK inhibitors.
