# Supplementary material for: Efficacy of BRAF and MEK Inhibition in Patients with BRAF-Mutant Advanced Melanoma and Germline CDKN2A Pathogenic Variants
Source: Cancers (Basel). 2021 May 18;13(10):2440. doi: 10.3390/cancers13102440 (PMC8157545; doi:10.3390/cancers13102440)
Supplement: Supplementary file 1 [file cancers-13-02440-s001.zip › cancers-1207401-supplementary.pdf]

Supplementary Table 1. Individualized data for the cohort of patients with germline *CDKN2A* pathogenic variants and *BRAF* V600 mutated metastatic melanoma treated with BRAF and MEK inhibitors included in our study.

ID	Region	Sex	Germline *CDKN2A* variant	Age at Tx start	AJCC 8th ed.	Baseline LDH	Treatment	Response	PFS *	OS*	Censor Date	Alive at censor Date	G3-4 AEs
ST2	Sweden	F	p.Pro81Ser	62	M1c	Normal	Dabra+Tram	PR	23	27+	Feb 2018	YES	NO
GO2	Sweden	M	p.Arg112dup	34	M1c	NA	Encor+Bini	PR	9	10	Mar 2016	NO	NO
BCN1	Barcelona	M	p.Gly101Trp	67	M1d	NA	Dabrafenib	PR	30	30	29/10/2013	NO	NO
BCN2	Barcelona	F	c.106delG	68	M1c	NA	Dabra+Tram	PR	11+	11+	06/01/2018	YES	NO
BCN3	Barcelona	F	c.-34G>T (homozygous)	43	M1d	NA	Dabra+Tram	PR	3	5	04/01/2018	NO	NO
BCN4	Barcelona	F	p.Arg124Cys	64	M1c	NA	Dabra+Tram+PDR001 (double blind clinical assay: CPDR001F2301)	PR	4	4+	15/01/2018	YES	NO
LE1	Leiden	M	19bp del	46	M1a	NA	Dabra+Tram	PR	3	4+	Jul 2017	YES	NO
LE2	Leiden	M	19bp del	62	M1c	NA	Vemurafenib	PR	3	4	Apr 2012	NO	NO
LE3	Leiden	M	19bp del	29	M1d	NA	Dabrafenib	PR	2	25+	Jul 2017	YES	YES
GE2	Genoa	F	G101W	57	M1c	Elevated	Dabra+Tram	PR	12	16	Oct 2016	NO	NO
GE3	Genoa	M	S56I	54	M1c	Elevated	Vemurafenib	PD	2	5	Sep 2015	NO	NO
GE6	Genoa	M	M1R	49	M1a	Normal	Dabra+Tram	PR	30+	30+	Aug 2019	YES	NO
GE8	Genoa	F	G101W	54	M1c	Elevated	Vemurafenib	PD	2	17	Apr 2016	NO	NO

GE9	Genoa	F	G101W	69	M1c	NA	Vemurafenib	SD	6	13	Jun 2015	NO	NO
GE10	Genoa	F	A68L	59	M1c	Elevated	Encor+Bini	PR	10	16	Mar 2017	NO	NO
GE11	Genoa	M	F90S	49	M1b	NA	Vemurafenib	PR	5	19	Mar 2015	NO	NO
GE12	Genoa	M	F90S	52	M1c	Elevated	Dabra+Tram	PR	5	9	Mar 2019	NO	NO
GE13	Genoa	M	E27X	69	M1d	Normal	Dabra+Tram	PR	9	19	May 2019	NO	NO
GE14	Genoa	F	G101W	60	M1d	Elevated	Dabra+Tram	PR	12+	12+	Sep 2019	YES	NO

Abbreviations: Bini= binimetinib; Dabra= dabrafenib; Encor= encorafenib; NA= not available; Tram= trametinib. *Survival rates with + prefix indicates that patient still has an ongoing PFS or OS

Supplementary Table 2. Summary of data from phase 3 clinical trials on patients with metastatic melanoma treated with BRAF and MEK inhibitors.

**Study**	**BRIM-3**[1, 2] **N= 337**	**BREAK-3**[3] **N=187**	**COBRIM**[4] **N=247**	**COMBI-d/COMBI-v**[5] **N= 563**	**COLUMBUS**[6] **N=192**
**Treatment**	Vemurafenib	Dabrafenib	Vemurafenib+cobimetinib	Dabrafenib+trametinib	Encorafenib+binimetinib
**Previous treatments**	Untreated	Untreated	Untreated	Untreated	Untreated and previously treated
**Age, years**					
**Median (range) [IQR]**	56[47–65]	53 (22–93)	56 (23–88)	55 (22–89)/ 55 (18–91)	57 [20–89]
**AJCC 7th edition stage, N (%)**					
M0	20 (6%)	6 (3%)	21 (9%)	19 (3%)	9 (5%)
M1a	34 (10%)	23 (12%)	40 (16%)	75 (13%)	26 (14%)
M1b	62 (18%)	34 (18%)	40 (16%)	105 (19%)	34 (18%)
M1c	221 (66%)	124 (66%)	146 (59%)	363 (64%)	123 (64%)
**Baseline LDH, N (%)**					
Normal	142 (42%)	119 (64%)	Not reported	365 (65%)	137 (71%)
Elevated	195 (58%)	67 (36%)	112 (46%)	194 (34%)	55 (29%)
Unknown	0 (0%)	1 (<1%)	Not reported	4 (1%)	0 (0%)
**Overall Response, N (%)**					
CR	19 (6%)	6 (3%)	39 (16%)	109 (19%)	37 (19%)
PR	173 (51%)	87 (47%)	133 (54%)	274 (49%)	108 (56%)
SD	Not reported	78 (42%)	44 (18%)	130 (23%)	33 (17%)
PD	Not reported	10 (5%)	19 (8%)	31 (6%)	14 (7%)

Not evaluable	Not reported	6 (3%)	12 (5%)	17 (3%)	0 (0%)
**Median progression-free survival**	6.9 months	5.1 months	12.3 months	11.1 months	14.9 months

Abbreviations: IQR= interquartile range

Supplementary Table 3. Summary of data from "real world" studies of patients with metastatic melanoma treated with BRAF and MEK inhibitors.

**Study**	**Vemurafenib safety study**[7, 8]	**Vemurafenib safety study (subanalysis of Italian population)**[9]	**Describe I**[10]	**Compassionate use of dabrafenib (retrospective analysis of Spanish population)**[11]	**Describe II**[12]	**Compassionate use of dabrafenib+trametinib (retrospective analysis of Spanish population)**[11]
**Number of patients**	3219	385	331	49	162*	87
**Treatment**	Vemurafenib	Vemurafenib	Dabrafenib	Dabrafenib	Dabrafenib+trametinib	Dabrafenib+trametinib
**Previous treatments**	Untreated and previously treated	Untreated and previously treated	Untreated and previously treated	Untreated and previously treated	Untreated	Untreated and previously treated
**Age, years**						
**Median (range) [IQR]**	55 (13-95)	NA	58 (18–86)	56.8 [26.2–84.3]	55.5 (22–87)	53.2 (24.8–81.3)
**AJCC 7th edition stage**						
M0	49 (2%)	4 (1%)	14 (4%)	6 (12%)	14 (9%)	0 (0%)
M1a	270 (8%)	48 (12%)	Not reported	7 (14%)	Not reported	12 (14%)
M1b	336 (10%)	53 (14%)	Not reported	8 (16%)	Not reported	19 (22%)
M1c	2562 (80%)	280 (73%)	Not reported	28 (57%)	Not reported	56 (64%)
**Brain metastases, n (%)**	753 (23%)	83 (22%)	132 (40%)	Not reported	62 (38%)	Not reported
**Baseline LDH, n (%)**		Not reported	Not reported	Not reported	Not reported	Not reported

Normal	1508 (47%)					
Elevated	1625 (50%)					
Unknown	86 (3%)					
**Overall Response**, n (%)						
CR	125 (5%)	14 (4%)	23 (7%)	7 (15%)	21 (13%)	18 (21%)
PR	865 (32%)	87 (26%)	129 (39%)	21 (45%)	88 (54%)	43 (51%)
SD	1399 (51%)	181 (54%)	67 (20%)	14 (29%)	20 (12%)	14 (17%)
PD	339 (12%)	43 (13%)	98 (30%)	5 (11%)	27 (17%)	9 (11%)
Not evaluable	9 (<1%)	7 (2%)	14 (4%)	Not reported	6 (4%)	Not reported
**Median progression-free survival**	5.6 months	5.9 months	5.2 months	8.1 months	7.5 months	10.2 months

*BRAF-inhibitor naive population only

References

1 McArthur GA, Chapman PB, Robert C, Larkin J, Haanen JB, Dummer R, Ribas A, Hogg D, Hamid O, Ascierto PA, Garbe C, Testori A, Maio M, Lorigan P, Lebbé C, Jouary T, Schadendorf D, O'Day SJ, Kirkwood JM, Eggermont AM, Dréno B, Sosman JA, Flaherty KT, Yin M, Caro I, Cheng S, Trunzer K, Hauschild A. Safety and efficacy of vemurafenib in BRAF(V600E) and BRAF(V600K) mutation-positive melanoma (BRIM-3): extended follow-up of a phase 3, randomised, open-label study. *The Lancet Oncology* 2014;**15**:323–332.

2 Chapman PB, Robert C, Larkin J, Haanen JB, Ribas A, Hogg D, Hamid O, Ascierto PA, Testori A, Lorigan PC, Dummer R, Sosman JA, Flaherty KT, Chang I, Coleman S, Caro I, Hauschild A, McArthur GA. Vemurafenib in patients with BRAFV600 mutation-positive metastatic melanoma: final overall survival results of the randomized BRIM-3 study. *Annals of oncology : official journal of the European Society for Medical Oncology* 2017;**28**:2581–2587.

3 Hauschild A, Grob J-J, Demidov LV, Jouary T, Gutzmer R, Millward M, Rutkowski P, Blank CU, Miller WH, Kaempgen E, Martín-Algarra S, Karaszewska B, Mauch C, Chiarion-Sileni V, Martin A-M, Swann S, Haney P, Mirakhur B, Guckert ME, Goodman V, Chapman PB. Dabrafenib in BRAF-mutated metastatic melanoma: a multicentre, open-label, phase 3 randomised controlled trial. *The Lancet* 2012;**380**:358–365.

4 Ascierto PA, McArthur GA, Dréno B, Atkinson V, Liszkay G, Di Giacomo AM, Mandalà M, Demidov L, Stroyakovskiy D, Thomas L, de la Cruz-Merino L, Dutriaux C, Garbe C, Yan Y, Wongchenko M, Chang I, Hsu JJ, Koralek DO, Rooney I, Ribas A, Larkin J. Cobimetinib combined with vemurafenib in advanced BRAFV600-mutant melanoma (coBRIM): updated efficacy results from a randomised, double-blind, phase 3 trial. *The Lancet Oncology* 2016;**17**:1248–1260.

5 Robert C, Grob JJ, Stroyakovskiy D, Karaszewska B, Hauschild A, Levchenko E, Chiarion Sileni V, Schachter J, Garbe C, Bondarenko I, Gogas H, Mandalá M, Haanen JBAG, Lebbé C, Mackiewicz A, Rutkowski P, Nathan PD, Ribas A, Davies MA, Flaherty KT, Burgess P, Tan M, Gasal E, Voi M, Schadendorf D, Long GV. Five-Year Outcomes with Dabrafenib plus Trametinib in Metastatic Melanoma. *New England Journal of Medicine* 2019;**381**:626–636.

6 Dummer R, Ascierto PA, Gogas HJ, Arance A, Mandala M, Liszkay G, Garbe C, Schadendorf D, Krajsova I, Gutzmer R, Chiarion Sileni V, Dutriaux C, de Groot JWB, Yamazaki N, Loquai C, Moutouh-de Parseval LA, Pickard MD, Sandor V, Robert C, Flaherty KT. Overall survival in patients with BRAF-mutant melanoma receiving encorafenib plus binimetinib versus vemurafenib or encorafenib (COLUMBUS): a multicentre, open-label, randomised, phase 3 trial. *The Lancet Oncology* 2018;**19**:1315–1327.

7 Blank CU, Larkin J, Arance AM, Hauschild A, Queirolo P, Del Vecchio M, Ascierto PA, Krajsova I, Schachter J, Neyns B, Garbe C, Chiarion Sileni V, Mandalà M, Gogas H, Espinosa E, Hospers GAP, Miller WH, Robson S, Makrutzki M, Antic V, Brown MP. Open-label, multicentre safety study of vemurafenib in 3219 patients with BRAFV600 mutation-positive metastatic melanoma: 2-year follow-up data and long-term responders' analysis. *European Journal of Cancer* 2017;**79**:176–184.

8 Larkin J, Brown MP, Arance AM, Hauschild A, Queirolo P, Vecchio MD, Ascierto PA, Krajsová I, Schachter J, Neyns B, Garbe C, Sileni VC, Mandalà M, Gogas H, Espinosa E, Hospers G, Lorigan P, Nyakas M, Guminski A, Liszkay G, Rutkowski P, Miller W, Donica M, Makrutzki M, Blank C. An open-label, multicentre safety study of vemurafenib in patients with BRAFV600-mutant metastatic melanoma: final analysis and a validated prognostic scoring system. *European Journal of Cancer* 2019;**107**:175–185.

9 Del Vecchio M, Ascierto PA, Mandalà M, Sileni VC, Maio M, Di Guardo L, Simeone E, Queirolo P. Vemurafenib in BRAFV600 mutated metastatic melanoma: a subanalysis of the Italian population of a global safety study. *Future Oncology* 2015;**11**:1355–1362.

10 Martin-Algarra S, Hinshelwood R, Mesnage S, Cebon J, Ferrucci PF, Aglietta M, Neyns B, Chiarion-Sileni V, Lindsay CR, Del Vecchio M, Linardou H, Merelli B, Tonini G, Atkinson V, Freivogel K, Stein D, Dalland L, Lau M, Legenne P, Queirolo P, Millward M. Effectiveness of dabrafenib in the treatment of patients with BRAF V600–mutated metastatic melanoma in a Named Patient Program. *Melanoma Research* 2019;**29**.https://journals.lww.com/melanomaresearch/Fulltext/2019/10000/Effectiveness_of_dabrafenib_in_the_treatment_of.10.aspx

11 Martín Algarra S, Soriano V, Fernández-Morales L, Berciano-Guerrero M-Á, Mujika K, Manzano JL, Puértolas Hernández T, Soria A, Rodríguez-Abreu D, Espinosa Arranz E, Medina Martínez J, Márquez-Rodas I, Rubió-Casadevall J, Ortega ME, Jurado García JM, Lecumberri

Biurrun MJ, Palacio I, Rodríguez de la Borbolla Artacho M, Altozano JP, Castellón Rubio VE, García A, Luna P, Ballesteros A, Fernández O, López Martín JA, Berrocal A, Arance A. Dabrafenib plus trametinib for compassionate use in metastatic melanoma: A STROBE-compliant retrospective observational postauthorization study. *Medicine* 2017;**96**:e9523–e9523.

12 Atkinson V, Sandhu S, Hospers G, Long GV, Aglietta M, Ferrucci PF, Tulyte S, Cappellini GCA, Soriano V, Ali S, Poprach A, Cesas A, Rodriguez-Abreu D, Lau M, de Jong E, Legenne P, Stein D, King B, van Thienen JV. Dabrafenib plus trametinib is effective in the treatment of BRAF V600-mutated metastatic melanoma patients: analysis of patients from the dabrafenib plus trametinib Named Patient Program (DESCRIBE II). *Melanoma Research* 9000;**Publish Ahead of Print**.https://journals.lww.com/melanomaresearch/Fulltext/publishahead/Dabrafenib_plus_trametinib_is_effective_in_the.99116.aspx
